# On the Importance of Being Ernst Mayr: “Darwin's apostle” died at the age of 100

**DOI:** 10.1371/journal.pbio.0030152

**Published:** 2005-04-05

**Authors:** Axel Meyer

## Abstract

Ernst Mayr lived for a century and accomplished more than several lifetimes worth of science in different biological disciplines, most notably evolutionary biology. Axel Meyer reflects on this remarkable man

Born on July 5, 1904, in Kempten in southern Germany, Ernst Mayr passed away peacefully at the Methuselah-like age of 100 on February 3, 2005, in Bedford near Cambridge, Massachusetts. Mayr was, by the accounts of his Harvard colleagues the late Stephen Jay Gould and Edward O. Wilson, not only the greatest evolutionary biologist of the 20th century, but even its greatest biologist overall. Thomas Henry Huxley was dubbed “Darwin's bulldog” for fighting for the acceptance of Darwinian ideas soon after their inception in the last decades of the 19th century. Similarly, Ernst Mayr has been called “Darwin's apostle” or the “Darwin of the 20th century” for promoting and dispersing Darwin's hypotheses throughout the past century.

Mayr lived for a century and accomplished more than several lifetime's worth of science in different biological disciplines. Brought up by parents who loved nature and who took the young Ernst on long hikes, he was exposed to natural history early on, but although birds were his passion all his life, he was, like Darwin, first compelled to study medicine. He began his studies at Greifswald—a prime birding spot—and through the chance observation of a rare species of duck that had not been seen in Germany for many years, he came in contact with the Berlin ornithologist Erwin Stresemann, who proposed that he switch to biology. Mayr abandoned medicine for biology and published his first scientific paper (of a total of almost 700) at the age of 19 in 1923, receiving his Ph.D. from Humboldt University in Berlin after only 16 months of graduate work and dissertation research; he was just 22. Ernst Mayr's last book (of a total of 25) was published in August 2004, a month after he turned 100 [1].

In 1931, thinking that he would not be offered a permanent post in Germany, he moved from Berlin to the American Museum of Natural History in Manhattan. In New York he called himself an ornithologist, and believed then, like many of his contemporaries, in Lamarkian inheritance. Sent by his advisor Erwin Stresemann from Berlin and financed by Lord Rothschild, he had just returned from over two years of perilous fieldwork in New Guinea and the Solomon Islands. The parallels to the lives of Darwin and Wallace may not be coincidental. During these expeditions, forlorn, at times given up for dead, exposed to tropical diseases and the danger of headhunters, he collected the skins of thousands of specimens, eating the flesh of many. Mayr was not only the ornithologist who probably tasted the largest number of different species of birds, but he also named 26 new species and over 400 new subspecies, more than any other taxonomist. In over 300 publications throughout his life, he discussed and described the geographic variation and distribution of birds and he also edited the last eight volumes of the *Checklist of the Birds of the World*. His main occupation during his 20 years at the American Museum was to curate and research the 280 000 specimens of the Rothschild collection.[Fig pbio-0030152-g001]

**Figure pbio-0030152-g001:**
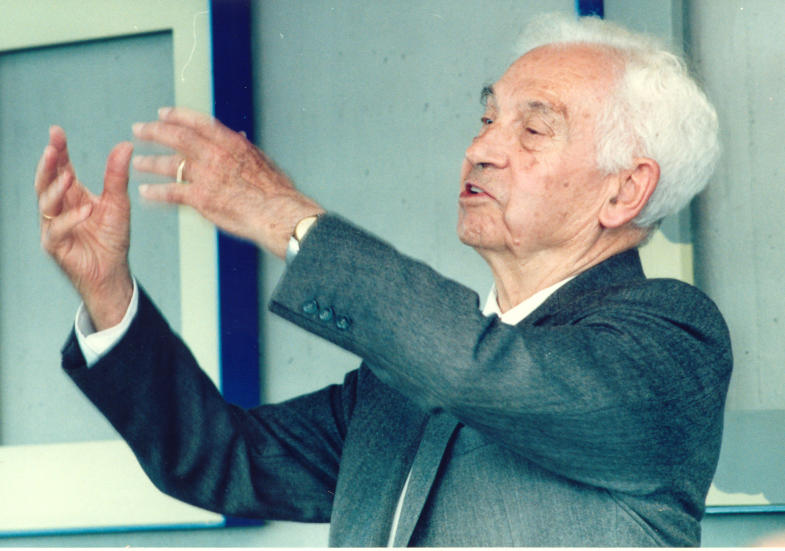
Ernst Mayr in 1994, after receiving an honorary degree at the University of Konstanz (Photo: University of Konstanz)

In the 1930s, Mayr's friendship and interactions with the Russian-born Columbia University population geneticist Theodosius Dobzhansky, author of the landmark text *Genetics and the Origin of Species* published in 1937 [2], started to influence his thinking. Mayr's interests subsequently began to diversify beyond taxonomy into evolutionary biology, and this expansion of his interests culminated in his first, and possibly still most important book, *Systematics and the Origin of Species*, published in 1942 [3]. This was his main contribution to the so-called Modern Synthesis of the 1930s and 1940s, a scientific sea change that came about largely through the contributions of Mayr and Dobzhansky and other scientists such as Ronald A. Fisher and George G. Simpson. Mayr's first book combined insights and methods from paleontology, population genetics, systematics, and natural history, thus providing a unified modern evolutionary theory. Patterns and processes in natural populations would now be seen as consistent with Darwinian natural selection and Mendelian mechanisms of inheritance, and the behavior of genes in populations came to be understood through laboratory population genetic experiments and theoretical mathematical predictions. Mayr was the last survivor, and historical eyewitness, among the architects of the Modern Synthesis.

Ernst Mayr had many fundamental insights into evolutionary biology, and almost every topic of importance in evolution was advanced by his ideas. Perhaps his most widely known contribution is to the current notion of what constitutes a species. Darwin did not think that species were real in the philosophical sense, but rather that they were the result of the human predilection to perceive discontinuity among continuously varying individuals. Most biologists nowadays disagree with Darwin's view of species, largely because of Mayr's “biological species concept”. Together with Dobzhansky, Mayr developed this definition of species “as groups of interbreeding populations in nature, unable to exchange genes with other such groups living in the same area” [2,3]. Barriers to gene flow between species—termed reproductive isolating mechanisms—keep biological species distinct through processes such as species-specific mate choice and hybrid sterility. Although there are theoretical and operational problems with the biological species concept (e.g., it does not apply to asexually reproducing organisms such as bacteria), it is still, by far, the most widely used species concept among the 20 or so competing definitions that have been proposed in the past several decades. Students of biology all over the world have memorized Mayr's definition of species for more than half a century.

The biological species concept made it possible to study how species arise, since the criterion of reproductive isolation provided a scientifically rigorous litmus test. The origin of species is a topic to which Darwin himself, in spite of the promising title of his famous book, did not say all that much. Mayr's understanding of the biogeographic distributions of bird species, overlaid with extensive knowledge about variation in morphology, led him to develop concepts about the geographic mechanisms of speciation—cornerstones for those studying speciation today. The geographic separation of populations, such as by rivers or valleys, he argued, prohibits homogenizing gene flow between them. If such isolated (termed allopatric) populations accumulate mutations over time, this might lead to the divergence of such populations from each other, and reproductive isolation might arise as a simple byproduct of these separate evolutionary histories. Mayr staunchly defended this idea during sometimes heated debates and further developed it and other hypotheses regarding geographic mechanisms of speciation over many decades (outlined in depth in the 797 pages of *Animal Species and Evolution* [4]).

One mechanism of speciation in particular is still contested (see [5] and [6]). Mayr called it “peripatric speciation” or “founder-effect speciation.” And it is an idea that Ernst Mayr was particularly fond of. He believed it to be his most important contribution to evolutionary biology. This model was again inspired by Mayr's own natural history observations. He noted that on some New Guinea islands, populations of birds differed markedly from individuals of the mainland population. He reasoned that this differentiation and speciation could result from a small number of individuals founding the island population. By bringing only a subset of all the genes of the main population (causing a genetic bottleneck), genetic drift (random fixation) and natural selection (due to a different set of selection pressures on these islands) would not only promote the formation of new species but would do so rapidly. This mechanism might also account for the paleontological pattern called “punctuated equilibrium,” which was proposed by Nils Eldredge and Gould in 1972 [7]. They noted that long periods of morphological stasis were sometimes interrupted (punctuated) by short periods of drastic phenotypic change in the fossil record. Somewhat ironically, Mayr, who considered himself a “gradualist” all his life, seems to have also provided a mechanism for variability in rates of evolution along evolutionary lineages.

After establishing the Society for the Study of Evolution and serving as the first editor of its journal, *Evolution*, Mayr moved to Harvard University in 1953 as the Alexander Agassiz Professor of Zoology and curator of birds at the Museum of Comparative Zoology. By this time, one surely would have labeled him primarily an evolutionary biologist rather than an ornithologist. His interests expanded even further into the theory of systematics—another field to which he made many contributions (see *Principles of Systematic Zoology* [8]). A lifelong and, it seems fair to say, futile fight with the then emerging idea of cladistics in systematic biology began. Ernst Mayr also served as director of the Museum of Comparative Zoology before his retirement in 1975 and oversaw the building of a new addition to the museum, whose library was renamed after him ten years ago.

What of his retirement? Mayr published 14 of his 25 books in the 30 years that followed after his official retirement. During the last two decades of his life, Mayr began to think and write more about the history and philosophy of biology. His most important work of this period was *The Growth of Biological Thought* [9], a monumental 974 pages. Here, and in later books and publications (he also founded the *Journal of the History of Biology*), he laid out why he thought that the philosophy of biology is an autonomous science that differs fundamentally from the philosophy of science, which, Mayr implied, was largely derived from physics. He argued that biology is a science that is based on historical contingency as well as on many unpredictable and coincidental factors that make it impossible to discover laws. Rules, not laws, are all that one will be able to find in biology.

Clearly, Ernst Mayr felt very strongly that he had something of importance to say to the world. And the world, not only in its scientific realms, seemed to think likewise. He received almost 20 honorary degrees from major universities, was a member of more academies than any other scientist before him, and received most of the prizes that could possibly be awarded to a biologist, including the Japan Prize, the Balzan Prize, and the Crafoord Prize, the “Nobel Prize for ecologists and evolutionary biologists.”

How could one person possibly fit so much into one lifetime, even such an astonishingly long one? For a start, he was a man of stringent self-discipline, who would get up with (or before) the birds, like a good ornithologist should. Writing (longhand or dictation) was done mostly in the mornings, and long walks were part of every day, as were extended periods of reading and corresponding with his colleagues. Just like Darwin, Mayr wrote thousands of letters minding the business of others, telling his fellow scientists what he thought of their work, praising them but also advising them on missed literature and new directions for further study. He did not like to be bothered with those other menial things that also belong to living on this planet, and, luckily for him, Gretel, his wife of 55 years, mostly took care of that part. So after her death ten years ago, when he was in his early 90s, he had to learn how to cook a hamburger for himself.

Ernst was gifted with an astonishing clarity of thought. Something that always impressed and humbled me was that the transcripts of his dictated manuscripts required very little further editing. Even Mayr's native-English-speaking competitors praised him, obviously a nonnative, for his lucid and clear writing style. Ernst also had the ability to store an astonishing amount of information drawn from many different sources—his memory was spectacular. His ability to synthesize ideas, combined with an amazing recall of natural history, an exact visual memory, and an overall wide scientific horizon, was awe-inspiring and, more than in anyone else I've ever met, produced a plethora of novel ideas. His vitality was also legendary. He would still climb trees in his mid-80s to inspect birds' nests, and he bought his last new car after having passed the age of 90, much to the astonishment of the car salesman, as he once told me.

Pulitzer Prize winner Natalie Angier from the New York Times once described Ernst Mayr in an interview as “opinionated and elitist, courtly and generous.” Ernst Mayr was all that. He was strong-willed, had little patience for people who had not done their homework or talked without having their natural history straight or their line of reasoning well thought out, and he could be damning in his judgment of the ideas of others. Yet he generously shared not only his thoughts, but also charitably donated a good portion of his salary and most of his significant prize money to causes such as the Nature Conservancy and to endow prizes for young evolutionary biologists.

Although Ernst Mayr lived only about a tenth of the 969 years that Methuselah is purported to have lived, he still accomplished much more than one might expect to get done, even in a 100 years. More important than his scientific output is the overarching influence he has had on the thinking of three or four generations of biologists that he was a contemporary of and interacted with. The scientific world lost a giant and an inspirational thinker. His doctoral advisor Stresemann once called Mayr a rising star. Nobody since Darwin shed the light of insight as bright over the firmament of evolutionary biology as Ernst Mayr did. His star has not stopped shining, and his ideas will continue to live on for generations of young evolutionary biologists to come.

On the occasion of his 100th birthday Mayr published an article in Science [10] looking back over eight decades of research in evolution that he closed with the following words: “The new research has one most encouraging message for the active evolutionist: it is that evolutionary biology is an endless frontier and there is still plenty to be discovered. I only regret that I won't be present to enjoy these future developments.”
